# Schizophrenia Risk Prediction: Using Genetics in a British South-Asian Cohort

**DOI:** 10.1192/bjo.2026.11152

**Published:** 2026-06-30

**Authors:** Izemnur Arican, Han Zhang, Andrew McQuillin, Nick Bass, Emma Magavern

**Affiliations:** 1 East London Foundation Trust, London, United Kingdom; 2 University College London, London, United Kingdom; 3 Queen Mary University of London, London, United Kingdom

## Abstract

**Aims:**

South Asians are the largest ethnic minority group in the UK. Despite having an elevated incidence of psychotic disorders compared to the European-ancestry majority, this group remains underrepresented in genomic research.

The heritability of schizophrenia (SCZ) is high, with around one quarter attributable to common genetic variants. Polygenic risk scores (PRS) summarise their combined contribution. The NHS 10-year plan prioritises implementation of population-based PRS to facilitate early identification of common diseases. PRS are derived from largely European-ancestry genome-wide association studies (GWAS), raising questions about the transferability of the SCZ PRS in ethnic minority populations.

This study aimed to examine the association between SCZ PRS, SCZ diagnosis, and antipsychotic prescribing in a South Asian cohort. Additionally, it aimed to compare PRS between individuals prescribed any antipsychotic and those prescribed injectable antipsychotics or clozapine as proxies for illness severity.

**Methods:**

We analysed data from Genes & Health, a community-based cohort with linked genetic and electronic health records for over 65,000 Pakistani and Bangladeshi individuals in the UK. PRS were derived from summary statistics from a largely European-ancestry SCZ GWAS. We examined associations between PRS, SCZ diagnosis, and antipsychotic prescribing, with secondary analyses of clozapine or injectable antipsychotic use.

**Results:**

SCZ PRS was associated with SCZ diagnosis (OR=1.51 per SD). However, SCZ PRS showed only a weak association with antipsychotic use in the full sample (OR=1.04 per SD).

SCZ diagnosis was strongly associated with antipsychotic prescribing (OR=28.4). Among those with SCZ, there was a stronger association between PRS and antipsychotic use (OR=1.45 per SD). Quintile analyses showed a pronounced extreme-risk pattern, with individuals in the highest PRS quintile having higher odds of antipsychotic use compared with those in the lowest quintile (OR=3.4). The PRS–antipsychotic association was modified by SCZ diagnosis (p=0.048). Among individuals with a diagnosis of SCZ and prescribed an antipsychotic, a higher PRS was associated with increased odds of clozapine or injectable antipsychotic use (OR=1.37 per SD).

**Conclusion:**

SCZ PRS derived from European-ancestry GWAS is associated with a diagnosis of SCZ in a South Asian ancestry cohort and higher PRS scores were associated with antipsychotic prescriptions. In those with a diagnosis of SCZ, higher PRS scores were also associated with clozapine or injectable antipsychotic use. The absence of directly comparable European-ancestry dataset limits inferences about relative performance across ancestries. The next stage will address this through UK Biobank analyses.

